# Supplementary material for: Improving diabetic retinopathy screening using artificial intelligence: design, evaluation and before-and-after study of a custom development
Source: Front Digit Health. 2025 Jun 19;7:1547045. doi: 10.3389/fdgth.2025.1547045 (PMC12222278; doi:10.3389/fdgth.2025.1547045)
Supplement: Supplementary file 2 [file Datasheet1.pdf]

Supplementary Material
Improving diabetic retinopathy screening using Artificial Intelligence: design, evaluation and before-and-after study of a custom development.

1. Requirements of HUN

Table 1 shows the requirements of the University Hospital of Navarre (HUN) for an AI tool for DR screening, together with their motivation.

May 26, 2025

Requirement	Motivation
**Req 1**. Generates DR screening proposals.	Support DR screening with a second medical opinion.
**Req 1.1**. Offers a data-level integration API. The entire screening proposal can be interpreted by the HIS.	Offer functionalities based on AI's proposals, workflow orchestration and AI monitoring.
**Req 1.2**. Grades DR according to the ICDR severity scale (1).	This is the DR grading standard being used in the hospital.
**Req 1.3**. Provides always a calibrated fundus gradability score, even if the image is gradable.	Configure a gradability threshold adapted to local imaging settings. Enable image quality monitoring and the implementation of functionalities based on fundus gradability.
**Req 1.4**. Grades DR in bad quality images, even if the eye fundus is not entirely gradable.	Identify and prioritize DR signs despite of imaging issues or preexisting diseases, as cataracts.
**Req 1.5**. Enables automatic generation of health records that reflect the AI's DR screening proposal and the final DR screening decision. These records are stored as part of the patient's medical history, signed, and sent to the primary care physician as a customized report.	Streamline the current DR screening task.
**Req 1.6**. Allows for override and revocation. The GP can override the AI's screening proposal at any time.	Patient security.
**Req 1.7**. Enables worklist ordering.	Prioritize most severe patients, and separate gradable and non-gradable fundus images for grading.
**Req 1.8**. Provides explainable grades. The AI is not a black box and incorporates an explanation for AI's referral proposals.	Ease AI proposal assessments and improve DR grader's sensitivity.
**Req 1.9**. Enables customizable workflow orchestration based on independent DR severity and image quality scores. This can be accomplished if the AI provides independent, calibrated outputs (2) for both DR severity and image quality. Therefore, decision points can be adjusted without developing a new version of the AI.	Adapt the image quality requirements based on seasonal factors and enable patient direct referrals adjusted to the work capacity of the second screening level.
**Req 2**. Improves fundus images.	Ease DR grading task. Minimize issues due to DR grader's eye fatigue and improper visualization.
**Req 3**. Complies with HUN's clinical pathway and infrastructure, supporting all its cameras, imaging protocols and the entire patient population.	Project feasibility. The ongoing DR screening program should be assisted by AI.
**Req 3.1**. Supports HUN's cameras: Topcon TRC-NW400, Carl Zeiss Visucam Pro NM and Visucam 224.	Do not exclude any imaging site from AI assistance.
**Req 3.2** Supports current imaging protocol, without disrupting it.	Ease AI adoption and enable a retrospective study.
**Req 3.2.1**. Analyzes macula and optic-disc-centered images, if present. These are the Early Treatment Diabetic Retinopathy Study imaging protocol (ETDRS) (3) fields 2 and 1, respectively.	Ensure a robust and adequate DR assessment (4; 5).
**Req 3.2.2**. Supports single-field studies (eyes with a single macula-centered fundus image).	Avoid too restrictive imaging requirements.
**Req 3.2.3**. Ignores composite images.	These images are used by DR graders, but are redundant because they are composed of macula and optic-disc-centered fundus fields.
**Req 3.3**. Supports current patient population, including Type II diabetics. It does not exclude patient groups.	Ethical reasons. It also eases retrospective and prospective studies, as no patient conditions need to be known.
**Req 3.4**. Can be integrated without a dedicated PACS for the AI system.	HUN already has a centralized, unified PACS. Parallel systems increase DICOM management complexity and impact on end users.
**Req 4**. Can be evolved to support HUN's new intended uses as the screening program changes.	Enable the expected evolution of the screening program.
**Req 4.1**. Supports pediatric DR Type I screening, or can be evolved to support it.	Democratize and expand DR screening.
**Req 4.2**. Allows reduction of current human supervision.	Improve cost-effectiveness.
**Req 4.3**. Supports new camera models or can be evolved to support them.	Enable device update for an improved image taking and clinical assessment.
**Req 5**. Generates data for oversight and monitoring.	Data drift and malfunction early detection.
**Req 6**. Enables batch processing of previous studies.	Enable a before-and-after comparative study.
**Req 7**. GDPR compliant.	Ethical and legal compliance (6). Ensures patient privacy.

Table 1: Requirements for an AI tool to support DR screening at HUN.

2. Commercial devices

Table 2 details how six Class IIA CE-marked devices meet the requirements of HUN (as of January 2024). We excluded products with insufficient little public information, those imposing a non-standard 45º field camera, and those self-certified as Class I medical devices[1]. The excluded devices are the following: Retinalyze, Optos AI, Remidio Medios DR, Ulma UMI DR, iHealthScreen iPredict DR and MONA.Health. Note that we used exclusively public information without contacting any vendor.

[1]According to the EU Regulation 2017/745 (7), any device placed on the market that generates information used to make diagnostic or treatment decisions should have at least a Class IIa CE mark (8).

	IDx-DR	EyeArt	Retmarker	OpthAI	RetCad	SELENA+
Product Version	v2.3-2022	v2.2-2023	Latest[1]	Latest[1]	v2.1-2023	Latest[1]
**Req 1**. Generates DR screening proposals	Yes	Yes	Yes	Yes	Yes	Yes
**Req 1.1**. Offers a data-level integration API	Yes[2]	Yes[2]	No	Yes	No	No
**Req 1.2**. Grades DR using the ICDR severity scale	Yes	Yes	No [5]	Yes	Yes	Yes
**Req 1.3**. Provides always a calibrated gradability score	No	No	No	No	Yes	N/A
**Req 1.4**. Grades DR in bad quality images	No	No	No	Yes	N/A	No
**Req 1.5**. Generates automatic health records	Yes [2]	N/A	No	Yes	No	No
**Req 1.6**. Allows for override and revocation	Limited [6]	Limited[6]	Limited[6]	Yes	Yes [12]	N/A
**Req 1.7**. Allows worklist ordering	Limited [2]	N/A[3]	No	Limited[14]	No[7]	No
**Req 1.8**. Provides explainable grades	No	No	No	Yes	Yes	No
**Req 1.9**. Enables custom workflow orchestration	Limited [2]	N/A	No	Limited[14]	No	No
**Req 2**. Improves fundus images	No	No	No	No	Yes	No
**Req 3**. Complies with clinical pathway and infrastr.	No	No	No	No	N/A	N/A
**Req 3.1**. Supports HUN's cameras	Limited [8]	Limited [8]	No	N/A	N/A	N/A
**Req 3.2** Supports current imaging protocol	Limited	Limited	Limited	Limited	Limited	Limited
**Req 3.2.1**. Analyzes macula and optic disc	Yes	Yes	Yes	No	Yes	N/A
**Req 3.2.2**. Supports single-field studies	No	N/A	N/A	Yes	N/A	Yes
**Req 3.2.3**. Ignores composite images	N/A	N/A	N/A	No	N/A	N/A
**Req 3.3**. Supports current patient population	No[9]	N/A[10]	N/A	No[4]	N/A	N/A
**Req 3.4**. Can be integrated without a dedicated PACS	Yes	Yes	N/A	No[13]	No[13]	N/A
**Req 4**. Can be evolved for new intended uses	No[11]	No[11]	No[11]	No[11]	No[11]	No[11]
**Req 4.1**. Supports pediatric DR Type I patients	No	No	Yes	N/A	N/A	N/A
**Req 4.2**. Allows reduction of current human supervision	Yes	Yes	Yes	Yes	N/A	Yes
**Req 4.3**. Supports new camera models	No	No	No	N/A	No	No
**Req 5**. Generates data for oversight	Yes	Yes	N/A	Yes	N/A	No
**Req 6**. Allows batch processing of studies	N/A	N/A	N/A	N/A	N/A	N/A
**Req 7**. GDPR compliant	Yes	Yes	N/A	N/A	Yes	N/A
	(9–14)	(15–21)	(22–25)	(26–28)	(29–32)	(33–35)

[1] We could not find any mentioned product version.
[2] Limited to None-or-Mild / More-than-mild / Vision threatening DR / Poor image quality. Image quality is not assessed independently from DR, and no scores are given for neither of them (11; 14).
[3] API is mentioned as a feature, but we could not find any details.
[4] OpthAI is contraindicated in patients with fundus opacities such as cataracts.
[5] Retmarker is based on red dot turnover rate.
[6] DR is not assessed if the software determines that the image is not gradable. If a non-gradable assessment is revoked, the clinician will not have any other help coming from the AI tool.
[7] Only using their software. DR/AMD/Quality scores can be used to order worklists.
[8] Only supports HUN's Topcon cameras.
[9] IDx-DR's exclusion criteria exclude some HUN patient groups, such as patients with any history of retinal surgery (12).
[10] A recent pivotal evaluation of EyeArt excluded some HUN's patient groups, such as patients with retinal surgery (19).
[11] We haven't found any information about the possibility of evolving this device to a new intended use for a particular customer, which requires renewing its commercialization authorization. Due to the difficulty involved, we assume this feature as highly improbable.
[12] OpthAI and RetCad give separate scores for image quality and DR grades, thus, the reviewer can revoke any of both assessments.
[13] OpthAI only works with the Evolucare PACS system. RetCad requires a propietary camera-PACS server system to function.
[14] OpthAI does not provide calibrated gradability scores.

Table 2: Comparison of Class II CE-marked devices' compliance with HUN's requirements.

3. Development project life cycle

Figure 1 shows the steps taken during each project phase. The tasks of the AI development team are shown on the left, and the work of HUN stakeholders is shown on the right. The hospital has guided the entire AI life-cycle from the start: it has been the source of the requirements, expectations, constraints, labels and clinical standards, taking project-level decisions at the end of each phase. In fact, decisions were always taken by the hospital at the most important control points. Kaur et al. (36) proposed these control points in their review for better AI control, following the ISO/IEC TR 24028:2020 Overview of trustworthiness in artificial intelligence (37).

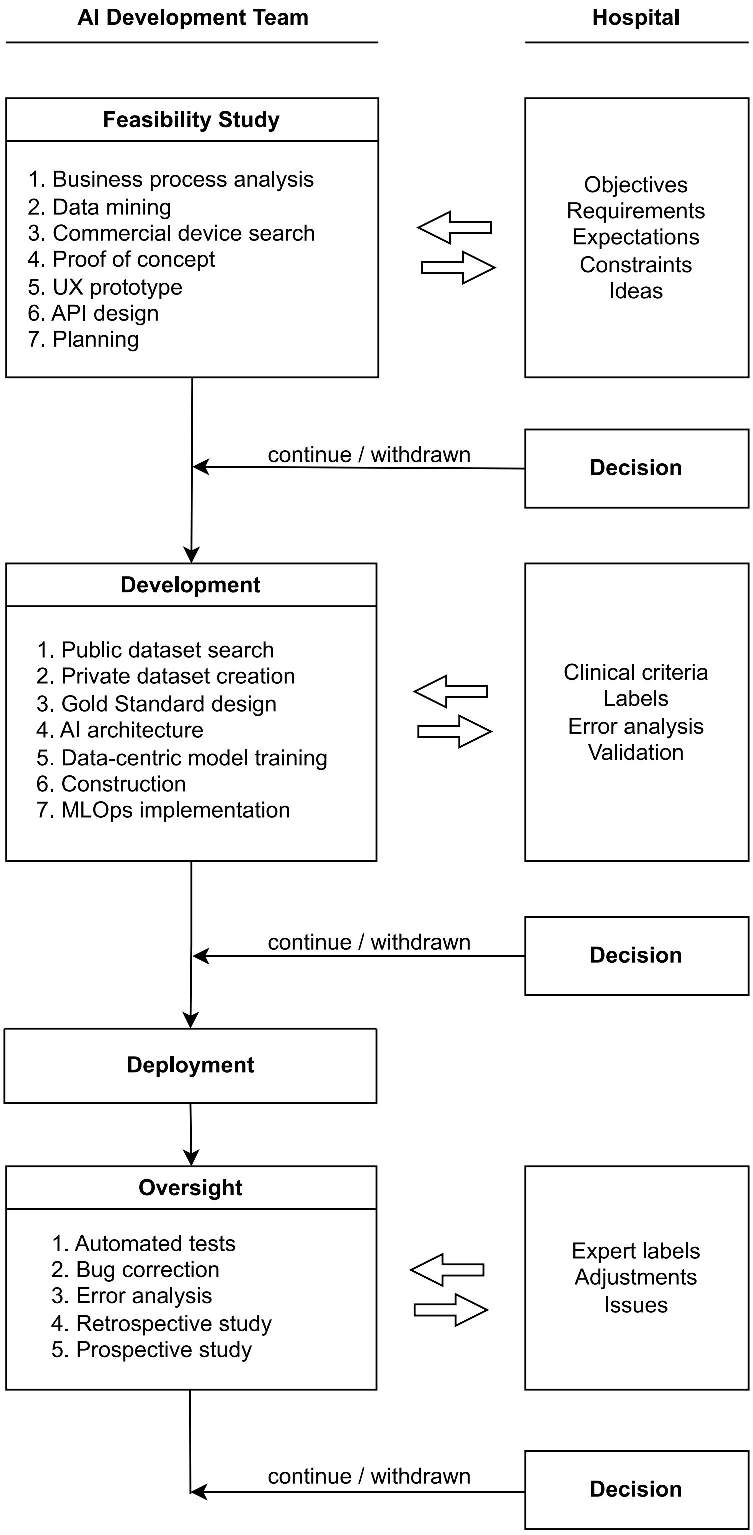

Figure 1: NaIA-RD project life cycle and involvement of the HUN Public Hospital. Note how the hospital guides the entire process and makes decisions at key control points.

4. Calibration

We randomly partitioned each DR and Gradability validation set 50 times (60% training and 40% testing) to choose a calibration algorithm that would generalize well. For each random partition, we trained and tested two different calibration algorithms: Isotonic Regression (38; 39), and Beta Calibration (40).

Beta Calibration showed better average results across partitions for the DR Classifier, and Isotonic Regression for the Gradability Classifier. Tables 3 (DR) and 4 (Gradability) show the results of these experiments, where the Estimated Calibration Error (ECE), Mean Calibration Error (MCE) and the Brier Score have been compared. In Figure 2, we show the reliability diagrams of a sample partition before and after calibrating with each method.

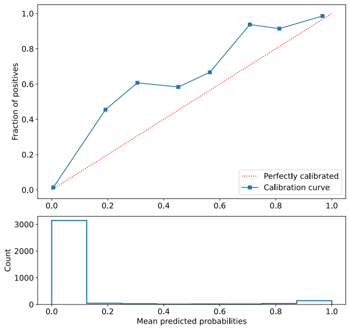

(a) DR Classifier: Uncalibrated.

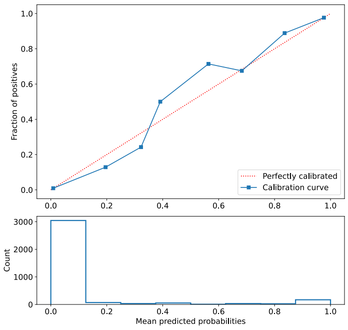

(b) DR Classifier: Isotonic calibration.

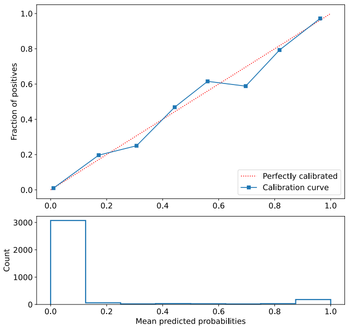

(c) DR Classifier: Beta calibration

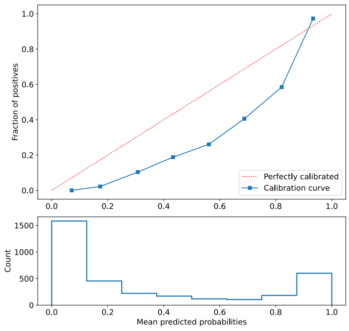

(d) Gradability Classifier: Uncalibrated

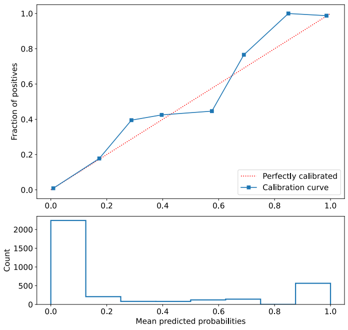

(e) Gradability Classifier: Isotonic calibration

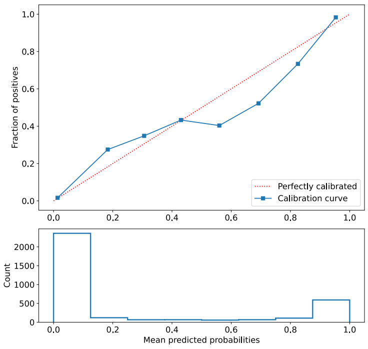

(f) Gradability Classifier: Beta calibration

Figure 2: Isotonic and Beta calibration comparison on a validation subset for DR Classifier (first row) and Gradability Classifier (second row). Calibration curves of the positive class are displayed along frequency histograms, grouped in 8 bins (we use 8 bins as they allow a correct visualization with enough detail), showing the calibration error. They compare predicted probabilities (x axis) with the proportion of actual positives in that bin (y axis). Calibrators are evaluated with data points that were excluded from their training sets.

Method	ECE	MCE	Brier Score
Uncalibrated	0.017248	0.299778	0.023504
Isotonic Regression	0.006349	0.214344	0.022356
Beta Calibration	0.005958	0.155110	0.021976

Table 3: DR Classifier's mean calibration metrics across 50 random partitions of the validation set.

Method	ECE	MCE	Brier Score
Uncalibrated	0.114798	0.278866	0.063906
Isotonic Regression	0.010228	0.116718	0.045477
Beta Calibration	0.017670	0.173586	0.046160

Table 4: Gradability Classifier's mean calibration metrics across 50 random partitions of the validation set.

5. API Design

NaIA-RD is designed as a RESTful microservice architecture, with the **Orchestrator** component exposing the main API endpoint for comprehensive diabetic retinopathy (DR) screening. This endpoint supports HTTP GET requests and is invoked by the Hospital Information System (HIS), which submits a list of fundus image URIs as query parameters. These URIs follow the WADO standard and point to DICOM studies stored as JPEG images on the hospital's PACS server(`GET /orquestator/screening?image_uri_list=<WADO_URI_1,...,WADO_URI_N>`).

The Orchestrator then sequentially invokes the REST APIs of the core microservices via internal HTTP GET requests:

1. It first calls the **Field Classifier** API (`GET /field_classifier/predict?image_uri=<WADO_URI>`) for each provided image URI. This service retrieves the JPEG image from the WADO URI and returns a classification, enabling the Orchestrator to identify the central and nasal fields.
2. It then uses the central field URI to call the **Gradability Classifier** API (`GET /gradability_classifier/predict?image_uri=<WADO_URI_central>`).
3. Finally, it calls the **DR Classifier** API (`GET /dr_classifier/predict?image_uri=<WADO_URI_field>`) for both the central and nasal field image URIs.

Each classifier microservice fetches the image from the specified WADO URI and returns a structured JSON response, indicated by the `Content-Type: application/json` header. The Orchestrator aggregates these responses, applies its decision logic, and returns a consolidated JSON object to the HIS. This response includes:

- The overall screening proposal (*referable* or *non-referable*), including its justification (*possible DR* or *non-gradability*), along with the corresponding DR and gradability scores,
- Fundus field, Gradability and DR classification results per image, including detailed scores,
- Product version,
- Detailed inference execution times,
- Two additional URIs for each image that has been classified as *possible DR*:
  1. An annotation endpoint on the **DR Classifier** (`GET /dr_classifier/annotations?image_uri=<WADO_URI>&type=integrated_gradients`). The default response is a JSON object with integrated gradients-based heatmap, structured for compatibility with DICOM graphic annotations. A JPEG image with the overlaid annotations can be retrieved using an `Accept: image/jpeg` header.
  2. An endpoint to retrieve an enhanced version of the image (`GET /dr_classifier/enhanced_image?image_uri=<WADO_URI>`), which returns the image in a media type `image/jpeg`.

The HIS uses these URIs to make subsequent GET requests to retrieve the annotations and enhanced image, then incorporates them into the original DICOM object using appropriate DICOM commands.

All HTTP GET requests also include the `accession_number` (study identifier) and `patient_id` (patient identifier) query parameters for traceability, which are provided by the HIS (omitted here for brevity). Each request is logged in Graylog, capturing response times, errors, and full input/output data.

Lastly, this microservice architecture supports direct access to each classifier's API (Field, Gradability, DR), allowing modular use for specialized tasks. An NGINX reverse proxy handles all incoming traffic, routing requests to the appropriate Docker container endpoints.

Note that all API calls in NaIA-RD are synchronous and stateless by design. This is a deliberate and straightforward architectural choice: since inference times are very short (on the order of milliseconds), it simplifies the integration logic on the HIS side. It also facilitates monitoring and support, as no temporary images or clinical data are stored. In fact, all the enhanced images and heatmaps can be easily reproduced via the logged URIs, without accessing the entire DICOM study. However, this approach is only viable when WADO resources can be retrieved quickly, as downloading these images is typically the slowest and most unpredictable part of the process.

References

[1] C. P. Wilkinson, F. L. Ferris, R. E. Klein, P. P. Lee, C. D. Agardh, M. Davis, D. Dills, A. Kampik, R. Pararajasegaram, J. T. Verdaguer, F. Lum, Proposed international clinical diabetic retinopathy and diabetic macular edema disease severity scales, Ophthalmology 110 (2003) 1677–1682. doi:10.1016/S0161-6420(03)00475-5.

[2] T. S. Filho, H. Song, M. Perello-Nieto, R. Santos-Rodriguez, M. Kull, P. Flach, Classifier calibration: How to assess and improve predicted class probabilities: a survey, Machine Learning 112 (2021) 3211–3260. doi:10.48550/arxiv.2112.10327.

[3] Grading Diabetic Retinopathy from Stereoscopic Color Fundus Photographs — An Extension of the Modified Airlie House Classification: ETDRS Report Number 10, ETDRS Report (4 1991). doi:10.1016/j.ophtha.2020.01.030.

[4] T. Y. Wong, J. Kang, H. Goh, C. Y. Cheung, S. S. Sim, P. C. Tan, G. Siew, W. Tan, Retinal imaging techniques for diabetic retinopathy screening, Journal of Diabetes Science and Technology 10 (2016) 282–294. doi:10.1177/1932296816629491.

[5] J. C. Lee, L. Nguyen, L. S. Hynan, P. H. Blomquist, Comparison of 1-field, 2-fields, and 3-fields fundus photography for detection and grading of diabetic retinopathy, Journal of Diabetes and its Complications 33 (12 2019). doi:10.1016/J.JDIACOMP.2019.107441.

[6] Regulation (EU) 2016/679 of the European Parliament and of the Council, https://data.europa.eu/eli/reg/2016/679/oj (2016).

[7] Regulation (EU) 2017/745 of the European Parliament and of the Council, https://eur-lex.europa.eu/legal-content/EN/TXT/HTML/?uri=CELEX:32017R0745&from=ES#d1e1058-1-1 (2017).

[8] U. J. Muehlematter, P. Daniore, K. N. Vokinger, Approval of artificial intelligence and machine learning-based medical devices in the USA and Europe (2015–20): a comparative analysis, The Lancet Digital Health 3 (2021) e195–e203. doi:10.1016/S2589-7500(20)30292-2.

[9] A. A. van der Heijden, M. D. Abramoff, F. Verbraak, M. V. van Hecke, A. Liem, G. Nijpels, Validation of automated screening for referable diabetic retinopathy with the IDx-DR device in the hoorn diabetes care system, Acta ophthalmologica 96 (2018) 63–68. doi:10.1111/AOS.13613.

[10] M. D. Abràmoff, P. T. Lavin, M. Birch, N. Shah, J. C. Folk, Pivotal trial of an autonomous AI-based diagnostic system for detection of diabetic retinopathy in primary care offices, npj Digital Medicine 2018 1:1 1 (2018) 1–8. doi:10.1038/s41746-018-0040-6.

[11] IDx-DR API User Manual. Software Version 1, 2018-08-31, https://uploads-ssl.webflow.com/5c118f855cb29ab026a90802/5c1934cba39674ba524b3dfe_ENG-DRAPIV2EU-S00140_API%20User%20Manual%20(1).pdf, accessed: 2024-01-22 (2024).

[12] IDx-DR: Indications for Use – EU, https://www.digitaldiagnostics.com/products/eye-disease/indications-for-use-eu/, accessed: 2024-01-22 (2024).

[13] IDx-DR official web page, https://www.healthvisors.com/idx-dr/, accessed: 2024-01-22 (2024).

[14] FDA: De Novo Classification Request for IDx-DR, https://www.accessdata.fda.gov/cdrh_docs/reviews/DEN180001.pdf, accessed: 2024-01-22 (2018).

[15] EyeArt official web page, https://www.eyenuk.com/en/products/eyeart/, accessed: 2024-01-22 (2024).

[16] FDA: De Novo Classification Request for EyeArt , https://www.accessdata.fda.gov/cdrh_docs/pdf20/K200667.pdf, accessed: 2024-01-22 (2020).

[17] K. Solanki, C. Ramachandra, S. Bhat, M. Bhaskaranand, M. G. Nittala, S. R. Sadda, EyeArt: Automated, High-throughput, Image Analysis for Diabetic Retinopathy Screening, Investigative Ophthalmology & Visual Science 56 (2015) 1429–1429.

[18] M. Bhaskaranand, C. Ramachandra, S. Bhat, J. Cuadros, M. G. Nittala, S. R. Sadda, K. Solanki, The value of automated diabetic retinopathy screening with the EyeArt system: A study of more than 100,000 consecutive encounters from people with diabetes, Diabetes Technology and Therapeutics 21 (2019) 635–643. doi:10.1089/dia.2019.0164.

[19] E. Ipp, D. Liljenquist, B. Bode, V. N. Shah, S. Silverstein, C. D. Regillo, J. I. Lim, S. Sadda, A. Domalpally, G. Gray, M. Bhaskaranand, C. Ramachandra, K. Solanki, E. S. Group, H. B. DuBiner, P. Genter, J. Graham, A. Johnson, G. Levy-Clarke, R. D. Pesavento, M. D. Sherman, B. T. Kim, G. B. Walman, H. K. Akturk, H. Joseph, P. Joshee, B. Trippe, J. M. Gilbert, B. A. Blodi, S. Reed, J. Reimers, K. Lang, H. Cohn, R. Shaw, S. Watson, A. Ewen, N. Barrett, M. Swift, J. Gornbein, Pivotal Evaluation of an Artificial Intelligence System for Autonomous Detection of Referrable and Vision-Threatening Diabetic Retinopathy, JAMA Network Open 4 (2021) e2134254–e2134254. doi:10.1001/JAMANETWORKOPEN.2021.34254.

[20] J. I. Lim, C. D. Regillo, S. R. Sadda, E. Ipp, M. Bhaskaranand, C. Ramachandra, K. Solanki, Artificial Intelligence Detection of Diabetic Retinopathy, Ophthalmology Science 3 (2023) 100228. doi:10.1016/j.xops.2022.100228.

[21] P. Heydon, C. Egan, L. Bolter, R. Chambers, J. Anderson, S. Aldington, I. M. Stratton, P. H. Scanlon, L. Webster, S. Mann, A. D. Chemin, C. G. Owen, A. Tufail, A. R. Rudnicka, Prospective evaluation of an artificial intelligence-enabled algorithm for automated diabetic retinopathy screening of 30000 patients, British Journal of Ophthalmology 105 (2021) 723–728. doi:10.1136/BJOPHTHALMOL-2020-316594.

[22] Retmarker official web page, https://www.retmarker.com/morescreening/, accessed: 2024-01-22 (2024).

[23] Retmarker white paper, https://www.retmarker.com/wp-content/uploads/docs/screening/RTM_whitepaper_Screening.pdf, accessed: 2024-01-22 (2024).

[24] L. Ribeiro, C. M. Oliveira, C. Neves, J. D. Ramos, H. Ferreira, J. Cunha-Vaz, Screening for Diabetic Retinopathy in the Central Region of Portugal. Added Value of Automated 'Disease/No Disease' Grading, Ophthalmologica. Journal international d'ophtalmologie. International journal of ophthalmology. Zeitschrift fur Augenheilkunde 233 (2014) 96–103. doi:10.1159/000368426.

[25] C. M. Oliveira, L. M. Cristóvão, M. L. Ribeiro, J. R. Abreu, Improved Automated Screening of Diabetic Retinopathy, Ophthalmologica 226 (2011) 191–197. doi:10.1159/000330285.

[26] OpthAI official web page, https://www.ophtai.com/en/, accessed: 2024-01-22 (2024).

[27] S. Matta, M. Lamard, P. H. Conze, A. L. Guilcher, V. Ricquebourg, A. A. Benyoussef, P. Massin, J. B. Rottier, B. Cochener, G. Quellec, Automatic Screening for Ocular Anomalies Using Fundus Photographs, Optometry and Vision Science 99 (2022) 281–291. doi:10.1097/OPX.0000000000001845.

[28] G. Quellec, M. Lamard, B. Lay, A. L. Guilcher, A. Erginay, B. Cochener, P. Massin, Instant automatic diagnosis of diabetic retinopathy (6 2019).

[29] RetCad official web page, https://retcad.thirona.eu/, accessed: 2024-01-22 (2024).

[30] C. Skevas, H. Weindler, M. Levering, J. Engelberts, M. van Grinsven, T. Katz, Simultaneous screening and classification of diabetic retinopathy and age-related macular degeneration based on fundus photos—a prospective analysis of the RetCAD system, International Journal of Ophthalmology 15 (2022) 1985. doi:10.18240/IJO.2022.12.14.

[31] RetCad 2.1 white paper, https://retcad.thirona.eu/wp-content/uploads/2022/12/RetCAD2.1.0_WhitePaper.pdf, accessed: 2024-01-22 (2024).

[32] S. Meredith, M. van Grinsven, J. Engelberts, D. Clarke, V. Prior, J. Vodrey, A. Hammond, R. Muhammed, P. Kirby, Performance of an artificial intelligence automated system for diabetic eye screening in a large English population, Diabetic Medicine 40 (2023) e15055. doi:10.1111/DME.15055.

[33] SELENA+ official web page, https://www.synapxe.sg/healthtech/health-ai/selena/, accessed: 2024-19-01 (2024).

[34] D. S. W. Ting, C. Y. L. Cheung, G. Lim, G. S. W. Tan, N. D. Quang, A. Gan, H. Hamzah, R. Garcia-Franco, I. Y. S. Yeo, S. Y. Lee, E. Y. M. Wong, C. Sabanayagam, M. Baskaran, F. Ibrahim, N. C. Tan, E. A. Finkelstein, E. L. Lamoureux, I. Y. Wong, N. M. Bressler, S. Sivaprasad, R. Varma, J. B. Jonas, M. G. He, C. Y. Cheng, G. C. M. Cheung, T. Aung, W. Hsu, M. L. Lee, T. Y. Wong, Development and validation of a deep learning system for diabetic retinopathy and related eye diseases using retinal images from multiethnic populations with diabetes, JAMA 318 (2017) 2211–2223. doi:10.1001/JAMA.2017.18152.

[35] A. W. A. Ta, H. L. Goh, C. Ang, L. Y. Koh, K. Poon, S. M. Miller, Two Singapore public healthcare AI applications for national screening programs and other examples, Health Care Science (2022). doi:10.1002/hcs2.10.

[36] D. Kaur, S. Uslu, K. J. Rittichier, A. Durresi, Trustworthy artificial intelligence: A review, ACM Computing Surveys (CSUR) 55 (1 2022). doi:10.1145/3491209.

[37] I. O. for Standardization, ISO/IEC TR 24028:2020 - Information technology — Artificial intelligence — Overview of trustworthiness in artificial intelligence (2020).

[38] B. Zadrozny, C. Elkan, Transforming classifier scores into accurate multiclass probability estimates, Proceedings of the eighth ACM SIGKDD international conference on Knowledge discovery and data mining (2002) 694–699doi:10.1145/775047.775151.

[39] M. P. Naeini, G. F. Cooper, Binary classifier calibration using an ensemble of near isotonic regression models, Proceedings. IEEE International Conference on Data Mining 2016 (2016) 360. doi:10.1109/ICDM.2016.0047.

[40] M. Kull, T. S. Filho, P. Flach, Beta calibration: a well-founded and easily implemented improvement on logistic calibration for binary classifiers, in: A. Singh, J. Zhu (Eds.), Proceedings of the 20th International Conference on Artificial Intelligence and Statistics, Vol. 54 of Proceedings of Machine Learning Research, PMLR, 2017, pp. 623–631.
